# Functional reorganization of memory processing in the hippocampus is associated with neuroprotector GLP-1 levels in type 2 diabetes

**DOI:** 10.1016/j.heliyon.2024.e27412

**Published:** 2024-03-03

**Authors:** Nádia S. Canário, Joana Crisóstomo, Carolina Moreno, João V. Duarte, Isabel C. Duarte, Mário J. Ribeiro, Beatriz Caramelo, Leonor V. Gomes, Paulo Matafome, Francisco P. Oliveira, Miguel Castelo-Branco

**Affiliations:** a Coimbra Institute for Biomedical Imaging and Translational Research (CIBIT), Institute of Nuclear Sciences Applied to Health, Portugal; b Faculty of Medicine, University of Coimbra, Portugal; c Department of Endocrinology, Centro Hospitalar e Universitário de Coimbra (CHUC), Coimbra, Portugal; d The Faculty of Science and Technology, University of Coimbra, Portugal; e Coimbra Institute of Clinical and Biomedical Research (iCBR), Faculty of Medicine and Center of Innovative Biomedicine and Biotechnology (CIBB), University of Coimbra, Portugal; f Champalimaud Research, Champalimaud Foundation, Lisbon, Portugal

## Abstract

Type 2 diabetes (T2D) often impairs memory functions, suggesting specific vulnerability of the hippocampus. In vivo neuroimaging studies relating encoding and retrieval of memory information with endogenous neuroprotection are lacking. The neuroprotector glucagon-like peptide (GLP-1) has a high receptor density in anterior/ventral hippocampus, as shown by animal models. Using an innovative event-related fMRI design in 34 participants we investigated patterns of hippocampal activity in T2D (n = 17) without mild cognitive impairment (MCI) versus healthy controls (n = 17) during an episodic memory task. We directly measured neurovascular coupling by estimating the hemodynamic response function using event-related analysis related to encoding and retrieval of episodic information in the hippocampus. We applied a mixed-effects general linear model analysis and a two-factor ANOVA to test for group differences. Significant between-group differences were found for memory encoding, showing evidence for functional reorganization: T2D patients showed an augmented activation in the posterior hippocampus while anterior activation was reduced. The latter was negatively correlated with both GLP-1 pre- and post-breakfast levels, in the absence of grey matter changes. These results suggest that patients with T2D without MCI have pre-symptomatic functional reorganization in brain regions underlying episodic memory, as a function of the concentration of the neuroprotective neuropeptide GLP-1.

## Article Highlights

• T2D impacts cognitive function emphazising the need to explore if memory regions benefit from endogenous neuroprotection.
• We investigated memory related hippocampal activity in T2D without cognitive deficits and its association with GLP-1 levels a
• We found evidence for a functional reorganization in T2D within the hippocampus, which was associated with GLP-1 levels.
• The findings suggest that pre-symptomatic hippocampus reorganization links to GLP-1 concentration changes

## 1 Introduction

Type 2 diabetes (T2D) is a complex metabolic disease associated with insulin resistance [1]. Its complications include brain dysfunction, as supported by animal [2] and human studies where memory impairment have been suggested, with an intriguing relation to Alzheimer's dementia (AD) [3].

The hippocampus is a core region involved in episodic memory [4], particularly in encoding and retrieval, being part of the Papez circuit [5]. This structure is functionally segregated along its longitudinal axis, and this is similar across rodents, monkeys, and humans [6] with highly specific ventral (anterior in humans) hippocampal networks [7,8] with dissociable roles in learning, memory, stress and emotional processing [8]. It has a complex vascularization system which is particularly vulnerable to vascular risk factors [9]. This vulnerability is corroborated by studies that have identified reduced grey matter volume (GMv) in this region in T2D [10,11]. Connectivity studies also suggest both altered functional and effective connectivity between the hippocampus and cortical brain regions [12,13]. Moreover, the hippocampus bears a high concentration of insulin receptors and glucose transporters, as well as receptors for neuroprotective incretins such as GLP-1 [14,15]. GLP-1 receptors phylogenetically dominate the anterior (corresponding to the ventral subdivision in rodents) part of the hippocampus [16].

The majority of the neuroimaging studies investigating brain dysfunction in T2D were morphometric studies and functional resting-state studies [13,17]. Task-driven functional magnetic resonance studies (BOLD-fMRI) in this condition are rarer. Nonetheless, a few studies on BOLD-fMRI were performed using working memory [18,19], decision-making [20] and speed discrimination tasks [21], all suggesting differences in the BOLD response in T2D. Fewer studies have address BOLD-related activity during an episodic memory task in T2D [22,23]. One study by Marder and colleagues [22] found reduced activation in both dorsolateral prefrontal cortex during an encoding task and reduced deactivation of the DMN during recognition. Another study by Wood and colleagues [23] showed changed activation patterns during an encoding task in a set of parietal and temporal regions. No differences in hippocampal activation were found in both studies. Here, we used an event-related design that allows for an estimation of the true hemodynamic response function (HRF), which is the only way to truly estimate neurovascular coupling in T2D [21,24].

GLP-1 has recently been shown to pass the blood-brain barrier [25]. The effects of this incretin in neuroprotection and neurogenesis have also been reported in preclinical studies [14]. GLP1 binding to hippocampal receptors promotes excitatory synaptic activity [14,26]. Its receptors are expressed in various regions of human, primate, and rodent brains in particular the hippocampus and they mediate neuroprotection [14,27]. GLP-1 analogues may decrease the formation of amyloid plaques, mitigate memory impairment, prevent synapse loss in the hippocampus, and restore synaptic plasticity in a mouse model of Alzheimer's disease (AD) [28,29]. They also seem to protect against impairment of hippocampal late-phase long-term potentiation induced by Aβ1-40 and spatial learning deficits in rats [30].

Given the evidence that hippocampus is a sensitive target for cognitive impairment, which is relevant in both T2D and neurodegenerative diseases like AD, it is important to investigate the neuronal correlates of episodic memory in T2D in relation to endogenous neuroprotection.

Here, we aimed to investigate neural activity differences, and their relation with the levels of the neuroprotector GLP-1, by directly estimating HRF, in hippocampus in T2D without mild cognitive impairment (MCI) and non-diabetic controls, using event-related encoding and retrieval memory conditions. Because T2D is a risk factor for dementia [31,32] we aimed to study possible pre-symptomatic manifestations prior to overt cognitive dysfunction. Indeed it is important to study neurodegeneration before the onset of clinical manifestations which would allow to understand earliest changes that occurs in the disease**.** Considering prior evidence [33,34], we expected larger differences in the encoding task. Given the putative role of the GLP-1 systems in memory function in animals [14,35], and in potential reducing the risk for Alzheimer's Disease [36] this motivated the focus on major neuroprotective neuropeptide.

## 2 Results

### 2.1 Behavioural results

Behavioural data showed that participants (n = 17 patients; n = 17 controls, after an initial pool of 41 participants) were overall performance matched. No between-group differences were found for dprime (sensitivity measure computed by subtracting the normalized hit rate to the normalized false-alarm rate) nor omissions. However, between-group comparisons found differences in reaction time for the “faces_recent condition (U = 77.00, p < 0.035) and two marginally significant differences for the faces_old (U = 82.00, p < 0.053) and places_old conditions (U = 82.00, p < 0.053). None of these survived for correction for multiple comparisons (Supplemental Table 1).

### 2.2 Imaging results

#### 2.2.1 Hippocampus-based between-group comparisons

We firstly performed a GLM in a hippocampus mask, testing for differences in activity patterns in T2D compared to controls, using an event related memory encoding and retrieval design, using faces, scenes and words. We further performed correlation analyses between a) the activity patterns with GLP-1 concentration in both pre and post-breakfast, b) the activity patterns with HBA1C levels and c) the activity patterns with grey matter fraction (GMf) of hippocampus.

**Fig. 1:**
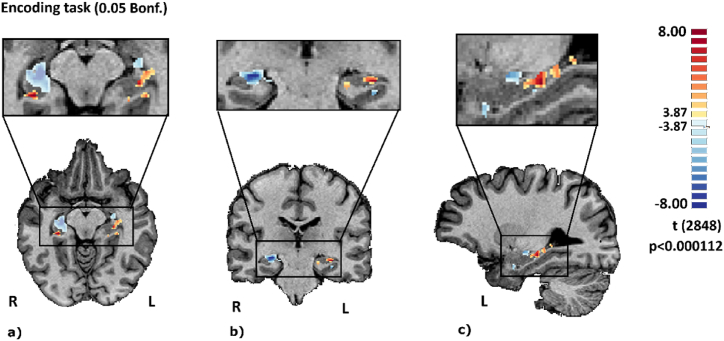
**Between-group differences in the hippocampus for the memory encoding task.** ROIs in the hippocampus depicting between-group comparisons in the encoding run, showing both positive and negative differences in clusters within in hippocampus. Panel (a) shows this negative/positive differences for anterior/posterior part of hippocampus, respectively, in a longitudinal perspective; (b) the same as before in a coronal view and (c) the same as before in a sagittal view.

For the fMRI encoding experiment (1st run), we found evidence for a distinct activity patterns in the T2D group. Accordingly, we found a differential pattern of response in the anterior hippocampus (3 ROIs with diminished activity, in the left hemisphere [2] and right hemisphere [1]), accompanied by an augmented activation in posterior hippocampus (3 ROIs, of which 2 in the left hemisphere and 1 in the right hemisphere) in the T2D group as compared to controls (see Table 1 and Fig. 1 for statistical maps of between-group differences). We used an operational definition of anterior and posterior hippocampus based on TAL y-coordinate equal or less than −20, as described in previous papers [37].

**Table 1:** Anatomical coordinates in TAL space according to ROIs' center of mass and peak voxel, *t*-test for the voxel with the most positive or negative difference between groups in that ROI and ROIs’ number of voxels. All ROIs were extracted from a bilateral hippocampus mask. We only included clusters ≥40 voxels.

ROIs' characterization extracted from a bilateral hippocampus mask					
Condition		Center of Mass	Peak Voxel	t	Nr. Voxels
Encoding	R/A	24,-20,-9	23,-19,-8	−12.44	617
	R/P	28,-31,-8	27,-31,-8	8.88	48
	L/A	−17,-12,-17	−15,-10,-18	−7.07	185
	L/P	−29,-35,-5	−30,-31,-11	7.45	250
	L/A	−26,-15,-8	−27,-13,-8	−5.96	57
	L/P	−29,-22,-10	−27,-25,-11	8.32	180

**Fig. 2:**
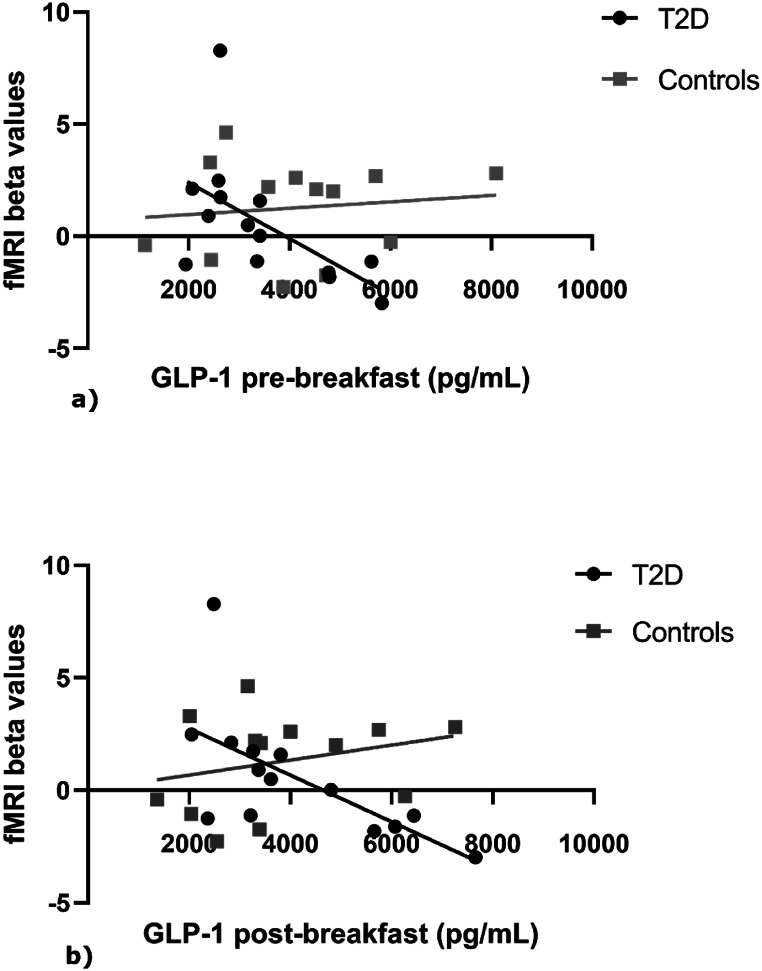
**Regression plots between GLP-1 and beta values of brain activation.** Linear regression plots comparing the association between GLP-1 levels in both pre (a) and post breakfast status (b) with beta values taken from the left anterior hippocampus. Figures depict both groups.

We found a strong negative correlation between GLP-1 concentration and the estimated effects observed in left anterior hippocampus in pre-breakfast status (pre: r = −0.635, p = 0.015). These results were replicated in the post-breakfast status in the T2D group (post: r = −0.688, p = 0.007). This effect was not seen in the control group (see Fig. 2) as confirmed by the direct slope group comparison of regression lines (GLP-1 at pre-breakfast, F [1,23] = 5.095, p = 0.033; GLP-1 at post-breakfast, F [1,23] = 7.477, p = 0.011;

These effects were specific to GLP-1, since no correlation was found between the estimated effects from all of the between-group differences ROIs and the HBA1C levels in both groups.

Mann-Whitney tests comparing the GMf across groups revealed no differences. No correlation between GMf and BOLD activity was found for both groups.

For the retrieval fMRI exploratory study (2nd run), we only found one cluster in posterior hippocampus, which showed a positive between-group difference, between the T2D group and controls (Supplemental Fig. 1). In this task two other clusters were found at right entorhinal cortex and in left amygdala. Also no between-group differences were found in GMf for the left posterior hippocampus ROI (U = 138.00, p = 0.823). Unlike the encoding task, no correlations were observed between GLP-1 concentration and brain activity. The same was true regarding HBA1C levels in both groups, and between GMf and BOLD activity.

Interestingly, we found a positive Spearman correlation between brain activity in right anterior hippocampus and disease duration (r = 0.601, p = 0.014), possibly suggesting an interaction between the effect of time and GLP-1 levels. As expected since participants from both groups were performance-matched, and therefore with a small range in performance values, no correlation was found between memory performance and activity patterns in hippocampus.

Results from whole-brain between-group comparisons are depicted in Supplemental Figs. 2 and 3.

## 3 Discussion

In this study we identified a distinct pattern of activity in the hippocampus in T2D without cognitive impairment, using an innovative event-related fMRI paradigm, allowing to directly estimate the neurovascular response. This response suggesting a functional reorganization in T2D was related to the levels of a neuroprotective neuropeptide which crosses the blood-brain-barrier, GLP-1. This neuropeptide is known to mediate direct neural effects in relation to memory functions [25,38]. Moreover, it does not change microvascular perfusion [25].

In particular, a distinct pattern of BOLD response in hippocampus in T2D was found compared with non-diabetic controls during dual memory encoding as well as in retrieval tasks. For the encoding task, overall between-group differences showed a higher response for T2D in posterior hippocampus and a lower response in a more anterior part of that region. Importantly, we found a strong negative correlation in T2D group between GLP-1 concentration and the estimated effects in left anterior hippocampus for the between-group differences analysis, which is consistent with the higher density of GLP-1R in the anterior hippocampus [16]. In the retrieval task, a higher response for the T2D group in left posterior hippocampus was also found. Our findings are consistent with models which posit anterior-posterior differentiation of function within the human hippocampal formation and complement other early steps toward a comparative (cross-species) model of the region [39].

The differential pattern of response found regarding the hippocampus axis (anterior/posterior) is striking considering the known distinct biological functions attributed to these sub-regions [33,40]. Previous animal models demonstrated that the dorsal part of the hippocampus (posterior hippocampus in humans) mediates cognitive functions such as spatial memory, navigation and memory retrieval whereas the more ventral part (homologous to the anterior hippocampus in humans) is more involved in emotional processing and memory encoding [40,41]. Furthermore, anatomical differences between the two regions in both input and output systems are well known [40,42]. Human studies also reiterate the distinction in terms of cognitive and functional properties of the hippocampus along the anterior/posterior axis [41,43].

Our findings are consistent with the hypothesis of an early functional disruption of the both anterior and posterior hippocampus in T2D. This may represent augmented compensatory activity, as a vicarious mechanism for weaker anterior encoding. Similar observations were found in the retrieval experiment.

The negative association found in T2D between the GLP-1 concentration and the estimated effects from the left anterior hippocampus region, is quite intriguing. We suggest that augmented levels of GLP-1 may represent a homeostatic response to reduced activation in this region. This compensatory mechanism may also be related to a likely reduction of GLP-1 receptors in this region as a consequence of pathophysiological changes. This would also help explain why lower brain activity is associated with higher GLP-1 levels. We believe this evidence is congruent with the neuroprotective effect of GLP-1 and we propose that in the presence of concomitant neuronal impairment GLP-1 levels may rise also to help maintain neuronal homeostasis. This might explain why we found a positive correlation between brain activity in disrupted right anterior hippocampus and disease duration, possibly suggesting a long term protective effect possibly derived from homeostatically increased GLP-1 levels. This is certainly a question to address in future work.

**Fig. 3:**
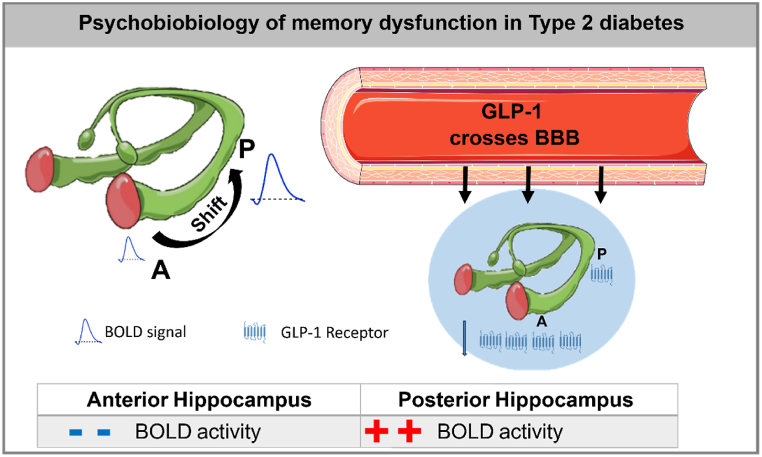
**Conceptual summary of main findings.** Left: we **found a distinct pattern of activity in anterior and posterior hippocampus in T2D during an** event related memory encoding responses (BOLD signal). Right: The anterior hippocampus is endowed with a high density of GLP-1 receptors, which may explain the homeostatic increase in GLP1 levels, a neuroprotector that crosses the Blood-Brain-Barrier.

Accordingly, GLP-1 as neuropeptide, passes the blood brain barrier and has specific receptors, namely GLP-1R, in many cerebral regions, such as hippocampus, and more concretely in CA1 sub region [14,26,38]. Acute elevation of plasma GLP-1 levels as seen postprandially can rapidly act on various regions of the brain to exert neurobiological actions [25]. One study addressed the relationship of between GLP-1 receptor activation (using receptor agonists) and brain activity in patients with T2D sample and it was performed using a food visualization task [44]. In that work, GLP-1 receptor activation by exenatide was associated with a response decrease in brain regions involved in appetite and reward processing, in obese participants with T2D. Lean healthy participants revealed no significant differences in brain response. Knockout of the GLP-1 receptor in mice impairs synaptic plasticity and memory [35]. Moreover, preclinical evidence also indicates that the neuroprotective role of GLP-1 is independent of glucose control [15]. This is consistent with the observed absence of association between HbA1C levels and brain activity. See Fig. 3 for a conceptual summary of our main findings.

Evidence of an altered memory-related neuronal response in T2D is consistent with human behaviour data [45,46] and animal studies [2]. Evidence associating T2D with higher risk of developing dementia [47] supports the idea of abnormal physiology in memory-related regions in this condition, since memory dysfunction is an important predictor of dementia [48]. Our samples were behaviourally matched at the group behavioural level but we were still able to find differences in BOLD activity, prior to cognitive impairment. This is evidence that functional abnormalities may precede cognitive deterioration [49]. Several imaging studies have suggested memory dysfunction to be associated with hippocampus volume [45,50] but here we did not observe differences attributable to GMf. Contrary to our study, participants in these works were not screened for MCI and the T2D sample had indeed decreased behavioural performances.

As far as we know no other study has found neither evidence for such a pattern of reorganization of the BOLD response in hippocampus in T2D nor a relation with GLP-1 levels. Furthermore, we were able to rule out vascular differences by directly calculating the hemodynamic response function by deconvolution. Previous fMRI studies revelled no differences in hippocampus in response to an episodic memory task [22,23]. We suggest that methodological limitations in the fMRI design of those studies might explain such results, namely the use of a block-design protocol instead of an event-related that allows to perform the deconvolution of the HRF. In fact, evidence of altered HRF in T2DM have been reported [21,24] taken as a strong argument for functional studies to adopt designs that allows to define HRF in this population by means of a deconvolution procedure in event-related experiments. Identifying possible early functional reorganization before the appearance of symptoms is of great importance because it allows us to understand the early effects of T2D in brain and for future longitudinal studies to explore the time it would take for cortical changes in T2D to manifest and whether a good management of T2D's metabolic state might prevent or mitigate overt cognitive impairment.

The present study has several limitations. One notable limitation is the relatively small sample size employed in this research. Future research should aim to include a more extensive participant pool to enhance the generalizability and robustness of the findings. Another limitation is the cross-sectional design. Longitudinal designs on the effects of T2D in brain and cognition are needed in order to understand how T2D might progress to progressive cognitive dysfunction and dementia.

In sum, the present study found evidence for early functional physiological anterior-posterior changes in the hippocampus in T2D, consistent with phylogenetically distinct subdivisions, associated with systemic GLP-1 levels in the absence of GM atrophy. Further molecular imaging research is necessary to determine the relationship between augmented peripheral GLP-1 levels and decreased GLP-1 hippocampal receptors.

## 4 Materials and methods

### 4.1 Resource availability

Lead contact: Professor M. Castelo-Branco (PhD)^.^ E-mail: mcbranco@fmed.uc.pt.

Materials availability: This study did not generate new unique reagents.

### 4.2 Participants

A total of 22 participants with T2D and 19 controls were initially enrolled. Five participants (3 with diabetes and 2 controls) were not able to complete the study due to claustrophobia, 1 (T2D group) due to psychiatric issues and 1 due to MCI (T2D group). After these exclusions, a total of 17 participants with T2D and 17 normoglycemic controls were enrolled in the fMRI experiment. The T2D group was diagnosed by the Endocrinology Department of Coimbra University Hospital Center using standard World Health Organization criteria [51]. Inclusion criteria were: 1) aged between 45 and 75 years old; 2) no history of central nervous system disorder, neoplastic nor inflammatory diseases; 3) no history of psychiatric diseases and 4) no history of current alcohol or drug abuse. We only included people with a T2D diagnosis for at least 1 year. All participants from the T2D group were on oral antidiabetic drugs and 11 were also taking insulin, 1 participant was taking GLP-1 receptor agonists and another participant was taking dipeptidyl peptidase-4 (DPP-4) inhibitor (these 2 participants were not included in the correlation analysis regarding the role of GLP-1).

The screening for the presence of amnestic MCI (aMCI) was based on clinical criteria of the National Institute of Neurological and Communicative Disorders and Stroke-Alzheimer's Disease and Related Disorders (NINCDS-ADRDA) [52]. The present study complied with the Declaration of Helsinki and was approved by the Ethics Committee of the Faculty of Medicine, University of Coimbra (approval number CE-041/2020). For more information regarding participant's demographic, clinical and cognitive variables see Table 2.

**Table 2:** Demographic, clinical and cognitive variables for all participants. The depicted biochemical variables were collected at fasting. Legend: M = mean; sd = standard deviation; Md = median; B.P = blood pressure; SMC = subjective memory complaints; GDS = geriatric depression scale; FCSRS = free and cued selective reminding test; RCF = Rey–Osterrieth complex figure; IAFAI = the adults and older adults’ functional assessment inventory; MoCA = Montreal cognitive assessment. Note1: HbA1C taken from 13 controls. Note2: Since FCSRT, RCF and IAFAI originated several dependent variables we performed a nonparametric multivariate test (MANOVA).

Demographic, clinical and cognitive variables for all participants			
	T2DM (M(sd); M_d_)	Controls (M(dp); M_d_)	*p*
*Age*	59.77(6.93); 61	58.94(8.07); 59	>0.729
*Education*	8.24(3.99); 8	9.24(4.68); 9	>0.561
*Gender (M;F)*	9; 8	10; 7	>0.999
*Duration of disease*	14 (6.63); 16	–	–
*HbA1C (%)*	8.62 (2.02); 8.70	5.44 (0.49); 5.50	**<0.001**
*Glucose (mg/dL)*	156.29 (56.10); 127.00	93.88 (14.07); 90.00	**<0.001**
*Insulin (mg/dL)*	27.97 (22.58); 22.00	12.99 (9.11); 9.22	**<0.030**
*GLP-1 pre-breakfast (pg/mL)*	3474.41 (1283.86); 3266.72	4175.16 (1820.39); 4124.74	**>0.243**
*GLP-1 post-breakfast (pg/mL)*	4116.08 (1726.00); 3490.67	3799.34 (1776.17); 3393.17	**>0.661**
*Total chol. (mg/dL)*	172.77 (44.27); 163.00	198.88 (56.60); 182.00	>0.143
*HDL (mg/dL)*	43.65 (11.45); 43.00	47.88 (11.64); 48.00	>0.292
*LDL (mg/dL)*	116.41 (36.82); 105.00	123.81 (47.91); 117.50	>0.620
*Triglic. (mg/dL)*	133.82 (60.71); 137.00	145.35 (79.09); 115.00	>0.808
*Height (cm)*	164.12 (9.10); 165.00	166.47 (7.53); 167.00	>0.417
*Weight (kg)*	79.53 (20.92); 82.10	79.49 (13.55); 78.40	>0.934
*IMC*	47.70 (10.60); 48.87	47.71 (7.40); 46.75	>0.997
*B.P. Systolic (mm Hg)*	141.94 (24.35); 148.00	142.00 (18.24); 139.00	>0.993
*B.P. Diastolic (mm Hg)*	80.10 (15.58); 76.00	85.65 (10.64); 85.00	>0.240
*Med – oral AntiD (%)*	100%	–	–
*Med – Insulin (%)*	65%	–	–
*Smok. habits (Yes;No)*	2; 8	3; 10	–
*Quit (Yes)*	7	4	–
*Alcoh. habits (Yes;No) (Yes;No)*	4; 13	8; 9	–
*Exerc. habits (Yes;No) (Yes;No)*	8; 9	9; 8	–
*SMC*	2.29(2.42); 2	1.88 (1.87); 2	>0.777
*GDS-30*	7.41(6.19); 6	5.41 (4.53); 4	>0.436
*FCSRT*			
*Total Immediate Recall*	42.12 (5.28); 43	41.35 (3.35); 42	>0.989
*Total Delayed Recall*	14.94 (1.14); 15	15.00 (1.00); 15	
*Free Recall Efficacy*	0.62 (0.13); 0.61	0.65 (0.09); 0.67	
*Total Recall Efficacy*	0.88 (0.11); 0.90	0.86 (0.07); 0.88	
*RCF*			
*Copy*	31.59 (3.44); 32	31.74 (3.40); 32	>0.994
*Immediate Recall*	16.41 (3.23); 16	17.65 (4.86); 17	
*Delayed Recall*	16.94 (3.56); 17	17.38 (4.96); 16	
*IAFAI*			
*Cognitive incapacity (%)*	0.12 (0.51); 0	0	>0.997
*Emotional incapacity (%)*	0.12 (0.51); 0	0	
*Physical incapacity*	1.74 (3.26); 0	0.12 (0.51); 0	
*MoCA (total score)*	23.82 (2.48); 24	24.53 (2.32); 24	>0.443
*Matrices (WAIS-III)*	11.77 (4.54); 12	14.24 (5.86); 15	>0.170
*Similarities (WAIS-III)*	18.35 (5.21); 18	19.65(23.74); 19	>0.396

### 4.3 Study design

All participants underwent blood collection at pre and post-breakfast periods and a physical examination was also performed by a physician at the Endocrinology Department of the Coimbra University Hospital Center, which included measurements of height, weight and resting blood pressure.

**Fig. 4:**
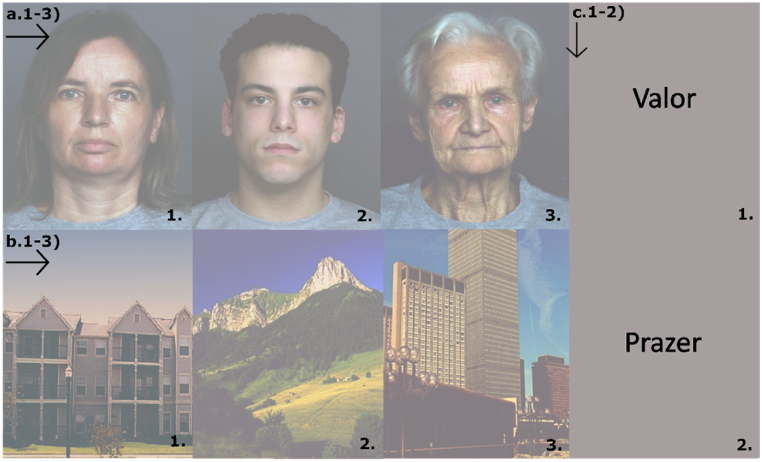
Images presented during the event-related fMRI task. From left to right: Middle, young and old faces (a); buildings, landscapes and skylines (b). We also depict images of words (c)included as verbal material in the experiment. All images are from a public database freely available for usage [54,55,58,59].

On the same day participants performed an event-related fMRI experiment, comprising two encoding and one retrieval tasks. The first encoding task was performed prior to scan, while the second encoding and the retrieval task were performed in the scanner. Overall, the three tasks shared the same design, and featured the presentation of three different visual categories - faces, scenes and words (Fig. 4). Face stimuli were randomly chosen by a pool of faces taken from the FACES database [53], and were composed of a balanced number of young, middle age and old faces. Scenes were taken from the database of the computational visual cognition laboratory [54] (http://cvcl.m it. edu/database.htm) and comprised a balanced number of skylines, buildings and landscapes. All images were taken from publicly available databases. Word stimuli were composed of frequent and low imageability words [55]. Word frequency was obtained in accordance with the Multifunctional Computational Lexicon of Contemporary Portuguese (MCLCP) from the Linguistic Center at the University of Lisbon. We choose verbal material with low imageability to prevent visual codification and thus evoke a strong left hemispheric response [56]. Furthermore, scenes and face stimuli were chosen to minimize a hemispheric bias in hippocampal response, considering evidence of the involvement of right hippocampus in face and scene processing [57]. Each category comprised 15 different coloured and luminance-matched stimuli presented in the center of a grey screen for 2 s, resulting in 45 stimuli presented to the subject. The correction of luminance was performed with SHINE toolbox. Average luminance was 66.2613 (+−1.5460) cd/m2. Each condition was followed by a baseline showing a cross of varied colour in the center of the screen. The baseline period was randomly defined between 4 different possible times – 4s, 6s, 8s, and 10s. This procedure of jittering the baseline condition was implemented along with the randomization of stimuli in order to avoid an overlapping of HRF at the same time points, thereby enabling deconvolution. For the two encoding tasks, each stimulus was repeated three times in order to enhance memory encoding. None of the stimuli presented in first encoding task was shown in the second encoding task (to ensure two memory time frames). In both tasks participants were instructed to do their best to memorize all the stimuli presented. The retrieval task had the same design as the encoding tasks, with the exception that, instead of having three repetitions per stimuli, participants saw a) new stimuli set as “new” condition, b) stimuli presented prior to the MRI task, defined as “old” memory condition, c) the same stimuli presented in the second encoding task (1st task inside scanner), defined as “recent” memory condition. Participants were instructed to press a particular MRI button for previously presented stimuli and another button for new materials. Baseline conditions also had a visual perception task, asking participants to press a button whenever the baseline cross would turn red or green. This procedure was implemented in order to divert participant's attention to this secondary task avoiding participants to retrieve previously presented stimuli during the baseline condition. Each task lasted for ∼21 min.

### 4.4 fMRI acquisition

Magnetic resonance imaging was performed on a 3 T S MAGNETOM Prisma Fit scanner (Siemens, Erlangen), equipped with a 64-channel birdcage head coil. Each session started with the acquisition of structural images by Magnetization‐Prepared Rapid Gradient Echo (MPRAGE) sequence with a voxel size of 1 × 1 × 1 mm^3^, and a field of view (FOV) of 256 × 256 mm^2^, acquiring a total of 176 **slices**. A repetition time (TR) of 2000 ms was set, an echo time (TE) at 3.5 ms and a flip angle of 7° was also applied along with an inversion time of 1100 ms. Structural images were followed by Echo Planar Imaging (EPI) sequences, each one lasting for approximately 21 min. The functional runs were acquired under the following parameters: TR = 2000 ms, TE = 30 ms, voxel size = 2.5 × 2.5 × 3 mm, FOV = 256 × 256 mm, and a flip angle of 90°. We acquired a total whole brain of 31 slices and 615 volumes. Before each functional run, a standard gradient field mapping (GRE) sequence was acquired in order to correct for field-inhomogeneity artifacts in EPI. Phase and magnitude field maps were acquired with a 3000 ms TR, TE of 30 ms, echo spacing of 0.5 ms, 100% phase resolution, phase encoding direction from anterior to posterior, echo time difference of 2.46 ms, and bandwidth in the phase direction of 14.21 Hz. Stimuli were displayed on an LCD monitor (NordicNeuroLab, Bergen, Norway) with a frequency rate of 60 Hz and placed approximately 156 cm away from the participant's head. The subject gave responses using a magnetic resonance-compatible joystick (Hybridmojo, San Mateo, CA, United States).

### 4.5 fMRI data pre-processing

Structural images were corrected for the inhomogeneity of signal intensity and oriented into the AC-PC plane and then transformed to the Talairach (TAL) reference system. Functional data were corrected for slice scan time and high-pass temporal filtered to remove low-frequency drifts and were corrected for signal intensity. We also applied a 3D motion correction, describing a translation/rotation along the x, y and z-axes and using sinc interpolation for the motion correction. fMRI data were first coregistered with anatomical scan in MRI native space and further transformed to TAL space using the structural images previously transformed to TAL reference system. The fMRI data coregistered to MRI space were already corrected for geometrical distortions using the AnatAbacus v1.1 plugin [60] for BrainVoyager 21.2 (Brain Innovation, the Netherlands).

### 4.6 Statistical analysis

We applied a random effects multistudy general linear model (RFX-GLM) analysis, using each participants deconvoluted time course, within a hippocampus mask, extracted from the Harvard-Oxford Cortical brain atlas (HOC) [61]. HOC atlas was transformed from Montreal Neurological Institute (MNI) to TAL space using GingerALE (3.0.2). The deconvolution of HRF was done to estimate the real HRF for each one of the 9 predictors from the two functional protocols, isolating the BOLD response for each data point. Because our clinical group was composed by people with diabetes, a population known to have potentially altered [21,24], the deconvolution method is critical since the shape of the HRF is not fixed in advance, and can be estimated. After running the RFX-GLM, we performed a two-factor ANOVA, with the Stimuli as the repeated measure factor and Group as the fixed factor in the analysis. For the encoding task (1st run) we sought for between-group differences accros stimulus types. For the recognition task (2nd run) we contrasted both “recent” and “old” conditions with the “new” condition to highlight brain responses to information retrieval rather than encoding. Nonetheless, we also performed an initial interaction analysis between the Conditions from the protocol and the Group (ConditionXGroup) to validate the use of all stimulus types in the between-group analysis. Since the differences in HRF parameters in the BOLD response curve in T2D (see Refs. [21,24] we obtained between-group statistical maps considering all points of the HRF curve. Both runs were corrected at the voxel level. The 1st run was Bonferroni corrected (<0.05). As an exploratory analysis, data from the 2nd run (only investigating retrieval) were corrected using FDR (<0.05). The latter experiment has an exploratory nature, as retrieval represents a comparatively less body of evidence regarding the involvement of the hippocampus, when contrasted with the robust support observed in encoding experiments.

We also performed a between-group comparison of GLP-1 levels using independent sample *t*-test and within-group analysis with the paired samples *t*-test. A Spearman correlation analysis was computed between this neuropeptide and the estimated brain activity effects (beta), from both RUNs, obtained in the between-group differences ROIs. For that, we considered the bilateral anterior and posterior hippocampus ROIs, as identified in the previous GLM approach done for the 1st run. Spearman correlation were also computed for the 2nd run using the left posterior hippocampus ROI identified in the GLM approach. Spearman correlation tests were further performed to correlate the estimated effects and HbA1C levels. A between-group analysis of grey matter fraction (GMf), in these ROIs was also performed, to control for regional atrophy. The extraction of GMf was performed using a customized MATLAB (R2017b) algorithm. For the GMf analysis we computed a Spearman correlation coefficient between ROIs estimated effects from the BOLD between-group analysis and the GMf from the respective ROIs.

A whole brain exploratory analysis is presented in supplemental material.

The behavioural performance in the fMRI retrieval task was compared by independent sample t tests or their nonparametric analogue. All significant between-groups results regarding behavioural performance and hormone effects were corrected for multiple comparisons using the Bonferroni correction.

Functional analysis of data was performed in BrainVoyager (Version 21.2, Brain Innovation, the Netherlands) and morphometric analysis was performed using the Computational Anatomy Toolbox 12 (CAT12), implemented within the Statistical Parametric Mapping 12 (SPM12), running in MATLAB (version R2017b, The Mathworks, MA). Both behavioural and correlation analysis was performed using IBM SPSS statistical package (v.27.0.1).

## Ethics declarations

This study was reviewed and approved by the local Research Ethics Committee at the Faculty of Medicine of Coimbra's University, with the approval number: CE-041/2020. All participants/patients provided informed consent to participate in the study.

## Data availability statement

Data will be made available upon reasonable request to the corresponding author.

## CRediT authorship contribution statement

**Nádia S. Canário:** Formal analysis, Investigation, Visualization, Writing – original draft, Writing – review & editing. **Joana Crisóstomo:** Visualization, Writing – review & editing. **Carolina Moreno:** Investigation, Validation, Writing – review & editing. **João V. Duarte:** Data curation, Validation, Writing – review & editing. **Isabel C. Duarte:** Formal analysis, Validation, Writing – review & editing. **Mário J. Ribeiro:** Investigation, Validation. **Beatriz Caramelo:** Resources, Validation, Writing – review & editing. **Leonor V. Gomes:** Resources, Validation, Writing – review & editing. **Paulo Matafome:** Methodology, Resources, Writing – review & editing. **Francisco P. Oliveira:** Investigation, Supervision, Validation, Writing – review & editing. **Miguel Castelo-Branco:** Conceptualization, Data curation, Funding acquisition, Investigation, Project administration, Resources, Supervision, Validation, Visualization, Writing – original draft, Writing – review & editing.

## Declaration of competing interest

The authors declare that they have no known competing financial interests or personal relationships that could have appeared to influence the work reported in this paper.
