# Supplementary material for: Functional reorganization of memory processing in the hippocampus is associated with neuroprotector GLP-1 levels in type 2 diabetes
Source: Heliyon. 2024 Mar 3;10(6):e27412. doi: 10.1016/j.heliyon.2024.e27412 (PMC10950584; doi:10.1016/j.heliyon.2024.e27412)
Supplement: Multimedia component 1 [file mmc1.docx]

|  |  |  | **New (M(sd)M_d_** | **Recent (M(sd)M_d_** | **Old (M(sd)M_d_** |
| --- | --- | --- | --- | --- | --- |
| **Dprime** | **T2DM** | Faces | 1.92(3.28)/1.73 | 2.47(2.68)/2.22 | 1.38(3.24)/0.43 |
|  |  | Places | 4.48(3.89)/3.00 | -0.05(1.35)/-0.17 | -0.77(1.63)/-0.86 |
|  |  | Verbal | 0.54(2.45)/1.26 | 0.70(2.33)/0.17 | 0.76(2.14)/0.60 |
|  | **Controls** | Faces | 2.29(3.24)/1.25 | 3.87(5.31)/3.00 | 2.75(3.94)/1.25 |
|  |  | Places | 3.82(3.68)/3.00 | 1.31(3.28)/0.51 | -0.14(1.19)/-0.17 |
|  |  | Verbal | -0.32(2.96)/-0.34 | 2.32(3.56)/1.47 | 2.10(3.19)/1.68 |
| **Omissions** | **T2DM** | Faces | 0.06(0.25)/0 | 0.06(0.25)/0 | 0.13(0.34)/0 |
|  |  | Places | 0.13(0.50)/0 | 0.19(0.54)/0 | 0.25(0.33)/0 |
|  |  | Verbal | 0.31(0.48)/0 | 0.38(0.89)/0 | 0.56(1.32)/0 |
|  |  | Faces | 0.18(0.39)/0 | 0.06(0.24)/0 | 0.18(0.39)/0 |
|  | **Controls** | Places | 0.35(0.79)/0 | 0.29(0.99)/0 | 0.35(0.79)/0 |
|  |  | Verbal | 0.88(3.14)/0 | 1.12(3.35)/0 | 0.94(3.13)/0 |
| **RT** | **T2DM** | Faces | 1.49(0.37)/1.43 | 1.43(0.33)/1.43 | 1.49(0.28)/1.47 |
|  |  | Places | 1.49(0.36)/1.42 | 1.54(0.30)/1.49 | 1.49(0.28)/1.47 |
|  |  | Verbal | 1.49(0.36)/1.50 | 1.49(0.35)/1.54 | 1.50(0.39)/1.51 |
|  | **Controls** | Faces | 1.40(0.23)/1.32 | 1.27(0.28)/1.21 | 1.32(0.06/1.35 |
|  |  | Places | 1.48(0.30)/1.52 | 1.42(0.23)/1.40 | 1.32(0.25)/1.35 |
|  |  | Verbal | 1.39(0.47)/1.36 | 1.47(0.38)/1.42 | 1.38(0.54)/1.40 |

**Supplemental table 1:** Descriptive statistics of dprime, omissions and RT for the retrieval task. Table depicts the results for both groups.


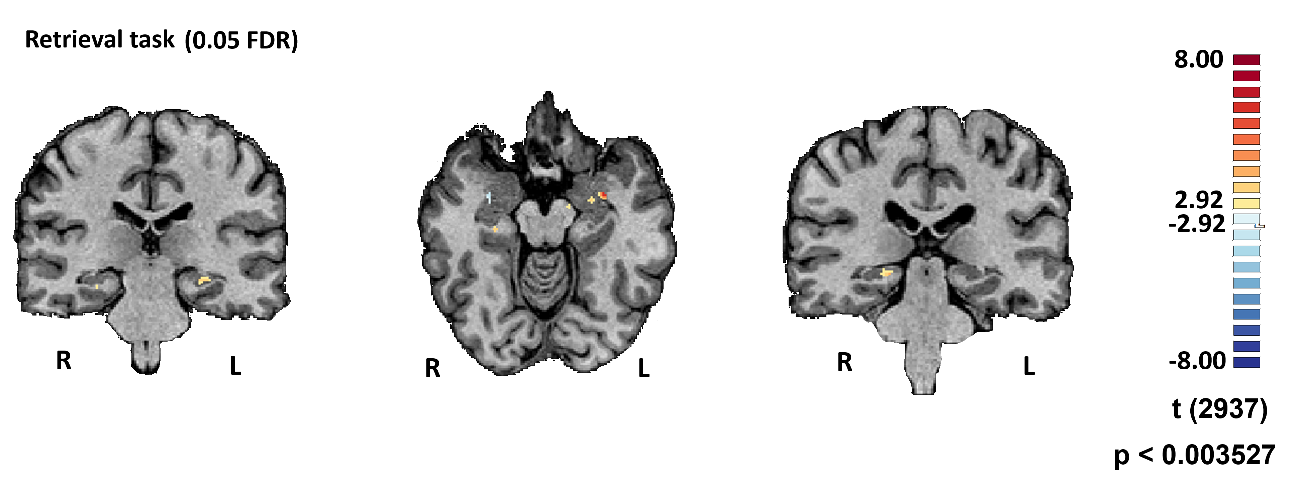


**Supplemental figure 1**: ROIs in the hippocampus depicting between-group comparisons in the retrieval task. Image depicts a total of 3 ROIs – left hippocampus (1^st^ image/ center of mass coordinates: -27, -24, -11), left amygdala (2^nd^ image/ center of mass coordinates: -30, -7, -14) and right entorhinal cortex (3^rd^ image/ center of mass coordinates: 20, -28, -8). This run included clusters ≥ 30 voxels.


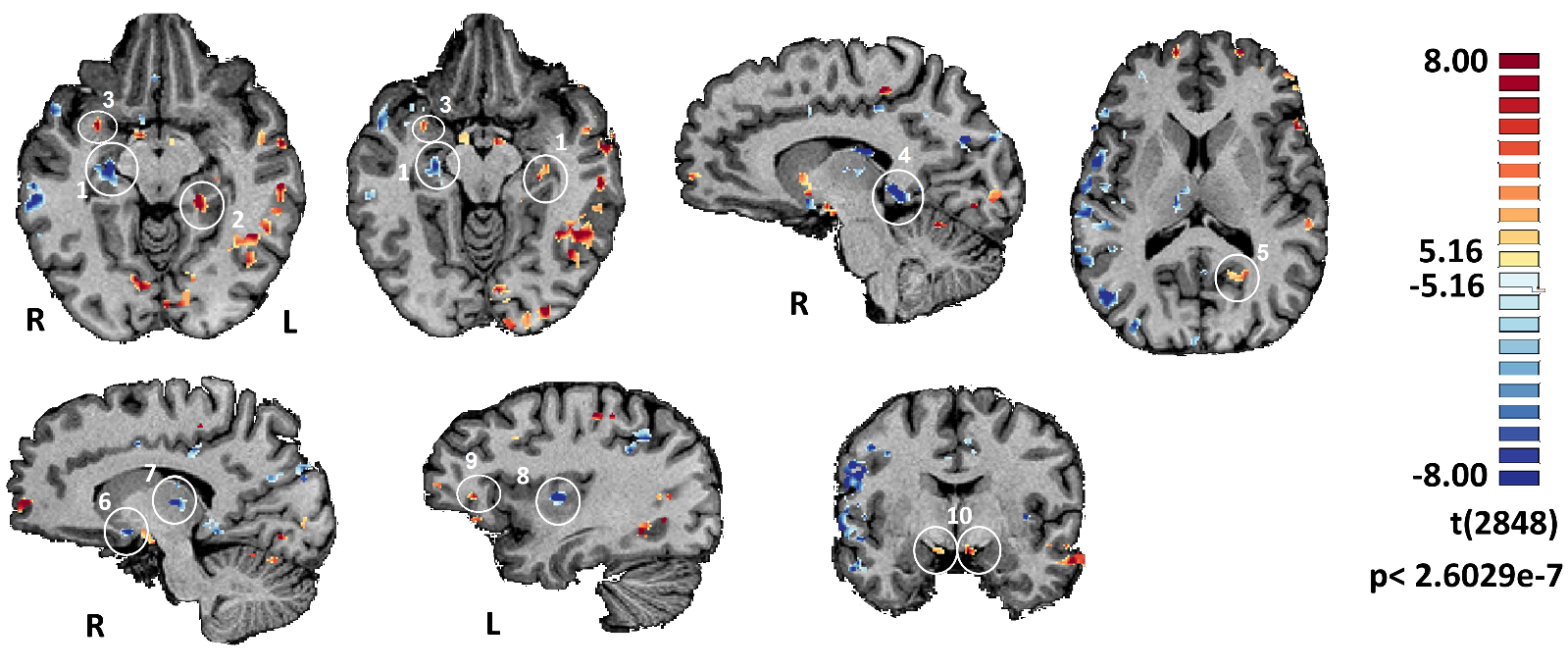


**Supplemental figure 2:** Whole-brain Bonferroni corrected (<0.005) map comparing T2D vs controls for the encoding task. The analysis was also performed using the same contrasts, and HRF’s data point as used in the hippocampus-based analysis. Legend: 1- right/left hippocampus; 2- left parahippocampal gyrus; 3- right entorhinal cortex; 4- right retroesplenial agranular cortex; 5- ventral part of left posterior cingulate cortex; 6- right accumbens; 7- right thalamus; 8- left insula; 9- left dorsolateral prefrontal cortex (BA10); 10- right/left mammillary bodies. Note: Only memory-related regions are identified.


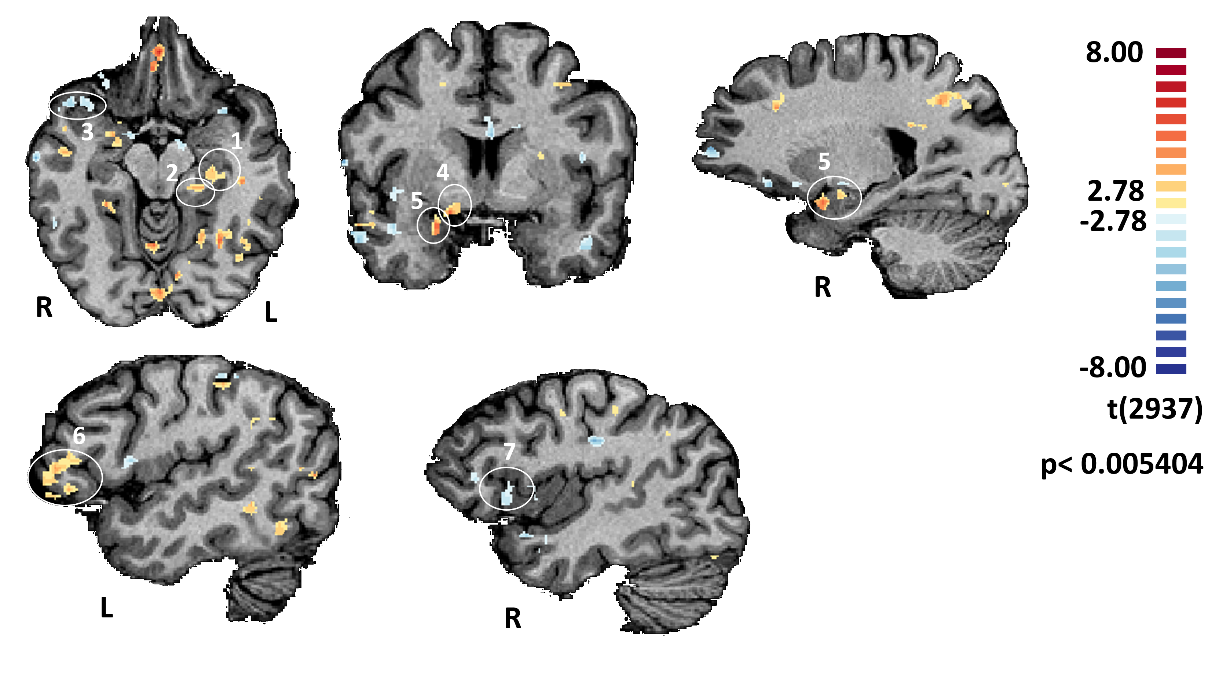


**Supplemental figure 3:** Whole-brain FDR corrected (<0.05) map comparing T2D vs controls for the retrieval task. The analysis was also performed using the same contrasts, and HRF’s data point, as used in the hippocampus-based analysis. Legend: 1- left hippocampus; 2- left parahippocampus gyrus; 3- right temporal pole; 4-right nucleus accumbens; 5- right amygdala; 6- left dorsolateral prefrontal cortex (BA46); 7- right amygdala. Note: Only memory-related regions are identified.
